# Draft Genomes of Six Wild Poisonous Mushrooms

**DOI:** 10.7150/jgen.75652

**Published:** 2022-08-01

**Authors:** Sittiporn Parnmen, Nattakarn Nooron, Sujitra Sikaphan, Chutimon Uttawichai, Dutsadee Polputpisatkul, Sriprapa Phatsarapongkul, Rungsaeng Chankunasuka, Unchalee Nitma, Chidkamon Thunkhamrak, Nisakorn Palakul, Khwanruan Naksuwankul, Onanong Pringsulaka, Achariya Rangsiruji

**Affiliations:** 1 Toxicology Center, National Institute of Health, Department of Medical Sciences, Ministry of Public Health, Nonthaburi 11000 Thailand.; 2 Department of Biology, Faculty of Science, Mahasarakham University, Mahasarakham 44150, Thailand.; 3 Department of Microbiology, Faculty of Science, Srinakharinwirot University, Bangkok 10110, Thailand.; 4 Department of Biology, Faculty of Science, Srinakharinwirot University, Bangkok 10110, Thailand.

**Keywords:** Poisonous mushroom, *De novo* genome assembly, Toxin-related gene, Mitogenome

## Abstract

Foodborne illnesses caused by wild mushroom poisoning occur globally and have led to food safety concerns. Here, we reported *de novo* genome assemblies of the six most commonly encountered toxic mushrooms in Thailand. These comprised *Amanita brunneitoxicaria*, *Cantharocybe virosa*, *Chlorophyllum molybdites*, *Entoloma mastoideum*, *Pseudosperma* sp. and *Russula subnigricans*. The nuclear genome sizes of these species ranged from 40 to 77 Mb, with the number of predicted genes ranging from 5,375 to 14,099. The mitogenome sizes varied from 41,555 to 78,907 bp. The resulting draft genomes of these poisonous mushrooms provide insights into toxin-related genes that may be used to establish genetic markers for monitoring mushroom poisoning outbreaks.

## Introduction

Foraging wild mushrooms is a popular leisure activity. However, due to their morphological resemblance, poisonous mushrooms are often misidentified as edible species. Unintentional ingestion of toxic mushrooms can result in adverse poisoning effects, ranging from mild gastrointestinal symptoms to severe cytotoxic effects and death of patients 1-5. Gastrointestinal irritant mushroom poisoning was most commonly encountered, followed by neurotoxic, cytotoxic, myotoxic and metabolic/endocrine toxicity mushroom poisoning 1. This study revealed the first report of draft genomes of six common poisonous mushrooms in Thailand. These included *Amanita brunneitoxicaria*, *Cantharocybe virosa*, *Chlorophyllum molybdites*, *Entoloma mastoideum*, *Pseudosperma* sp. and *Russula subnigricans*. According to the classification of mushroom toxicity by White et al. 6, these mushrooms cause five major types of toxicity, including cytotoxicity (*A. brunneitoxicaria*, D346), neurotoxicity (*Pseudosperma* sp., D523), myotoxicity (*R. subnigricans*, D338), metabolic/endocrine toxicity (*C. virosa*, D287), gastrointestinal toxicity (*C. molybdites*, D392), and gastrointestinal toxicity coupled with neurotoxicity **(***E. mastoideum*, D322**)**. Although ingestion of most of these mushrooms resulted in mild poisoning symptoms 4,5,7, *A. brunneitoxicaria* and *R. subnigricans* caused fatal mushroom poisoning 1,3.

## Materials and Methods

Mushroom samples were identified based on morphological and chemical characteristics, as well as ITS sequences 2-5. Genomic DNA was extracted from fruiting bodies using the DNeasy^TM^ Plant Mini Kit (QIAGEN, Germany). DNA concentrations of the samples were measured using fluorescence-based Qubit^TM^ quantitation assays (Qubit^®^ 2.0 Fluorometer, Invitrogen, USA). Paired-end libraries (2 x 150 bp) were prepared using the TruSeq Nano DNA Kit (Illumina^®^, USA) and subsequently sequenced on the NovaSeq 6000 platform (Illumina^®^, USA). Paired-end reads were quality filtered using BBDuk [qtrim=rl, trimq=20, minlength=30], and normalized read coverage was obtained using BBNorm [target=40, mindepth=6] (http://jgi.doe.gov/data-and-tools/bb-tools/). The filtered paired-end reads were assembled using SPAdes V 3.15.2 with different k-mers 8. Completeness evaluation of the final assemblies was conducted using BUSCO version 5.5.2 9. Eukaryotic gene prediction was carried out using AUGUSTUS version 3.4.0 10,11 with parameters trained from *Coprinopsis cinerea*. The orthologous gene clusters were compared using OrthoVenn2 12. Fungal metabolite biosynthetic gene clusters were analyzed using antiSMASH 6.0 13,14. Circular mitochondrial contigs were aligned using BLAST against the Nr database (http://www.ncbi.nlm.nih.gov/blast).

## Results and Discussion

**Figure 1:**
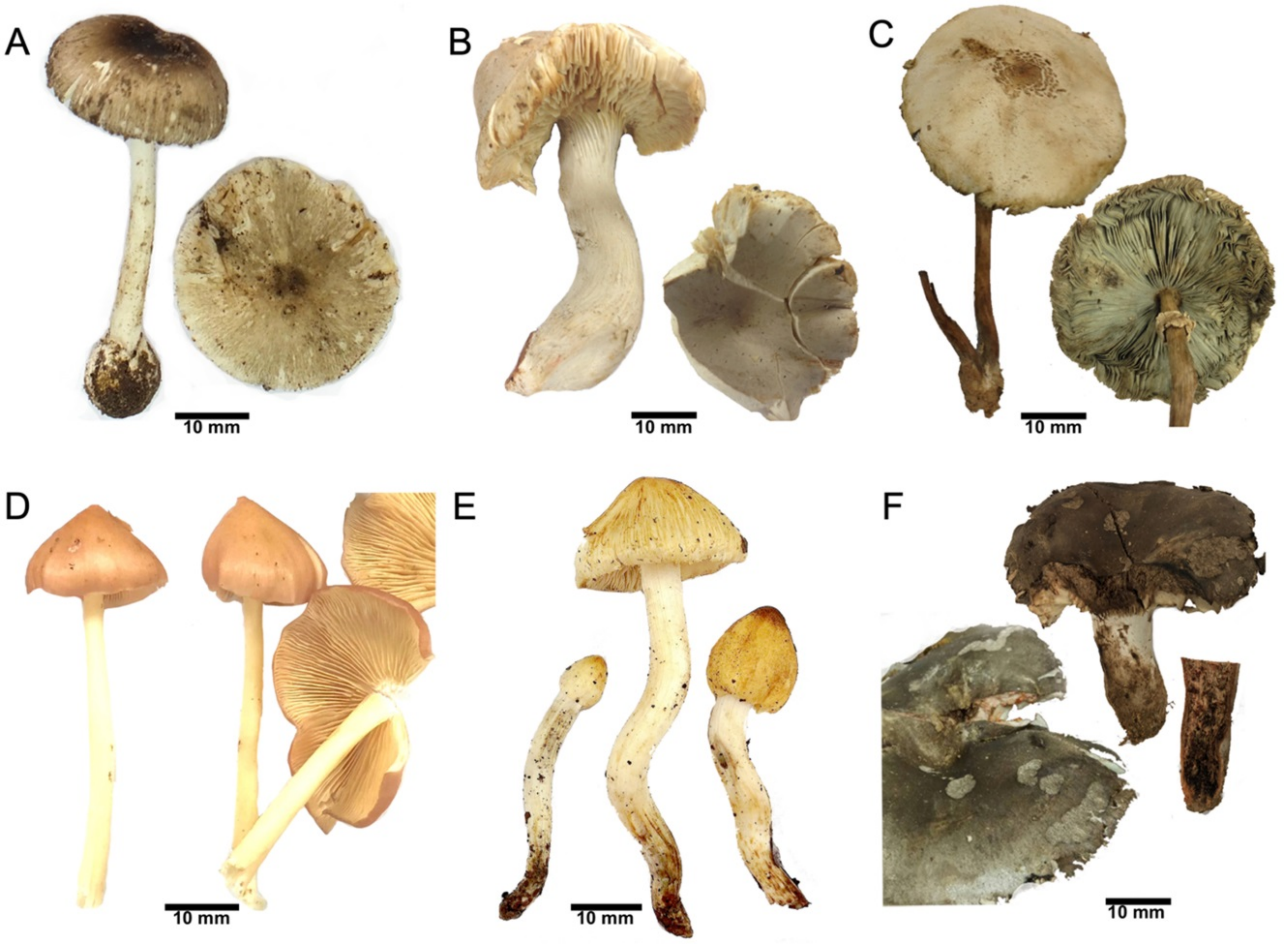
Mushroom samples. (**A**) *A. brunneitoxicaria* (D346), (**B**)* C. virosa* (D287), (**C**) *C. molybdites* (D392), (**D**) *E. mastoideum* (D322), (**E**) *Pseudosperma* sp. (D523) and (F) *R.* subnigricans (D338).

Six mushroom samples (Fig. 1) obtained from mushroom poisoning cases were left from food preparation. Detection of toxins revealed the presence of hepatotoxic amatoxins in *A. brunneitoxicaria* 3, and lethal cycloprop-2-ene carboxylic acid in *R. subnigricans* 15, 16.

**Figure 2:**
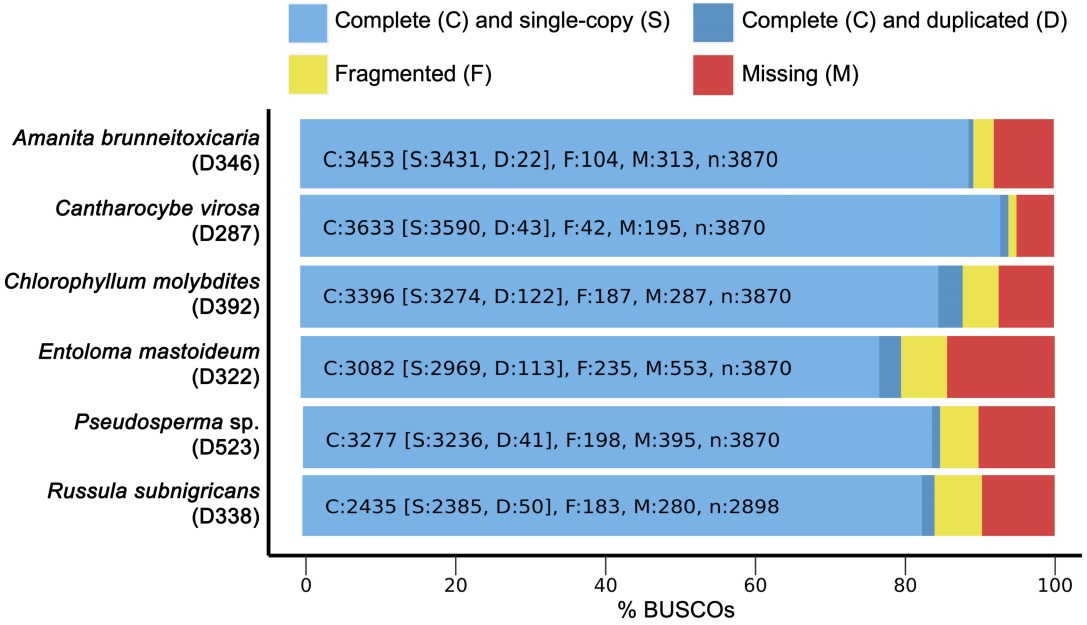
BUSCO completeness analysis for genome assemblies of six poisonous mushrooms.

**Figure 3:**
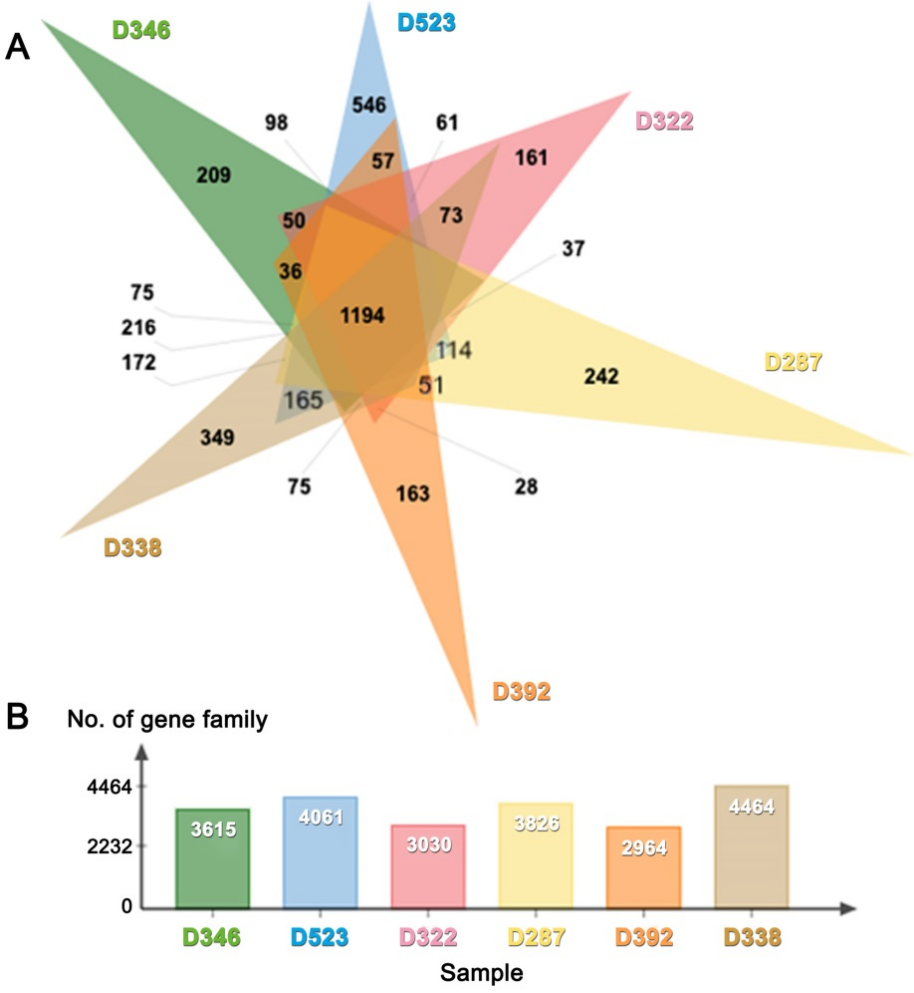
(**A**) Venn diagram and (**B**) bar graph showing the number of gene families present in six poisonous mushrooms.

*De novo* draft genome assemblies revealed the median read depth ranging from 82x to 118x. The coverage of genomes ranged from 97x to 115x. The longest scaffold length (800,654 bp) was present in *C. molybdites.* Details of the genome features of the six poisonous mushrooms are shown in Table 1. The complete BUSCO scores ranged from 84% to 95.7% (Fig. 2). The number of protein-coding genes predicted ranged from 5,375 to 14,099. All six mushroom species possessed 6,796 gene clusters, including 6,045 orthologous gene clusters and 751 single-copy gene clusters. The Venn diagram showed 1,194 shared gene families (Fig. 3). Of the 68 biosynthetic gene clusters obtained, toxin-related genes have been the focus of the current study. The terpene synthase gene cluster was abundantly found in all mushroom genomes. This gene family corresponds to the largest group of secondary metabolite products in fungi 17. The six mitogenomes ranged in size from 41,555 to 78,907 bp. For *R. subnigricans*, the size of mitogenome (66,439 bp) and GC content (21.6%) were similar to those reported in a previous study 18. The data obtained from the resulting draft genomes of these poisonous mushrooms pave the way for the development of effective genome-based diagnostics for clinical application through in-depth genomic research.

### Nucleotide Sequence Accession Numbers

Genome sequences were uploaded as BioProject **PRJNA834754**. The raw data were deposited in the NCBI/SRA database under the accession numbers **SAMN28055954** (*A. brunneitoxicaria*, D346), **SAMN28055957** (*C. virosa*, D287), **SAMN28055958** (*C. molybdites*, D392), **SAMN28055959** (*E. mastoideum*, D322), **SAMN28055955** (*Pseudosperma* sp., D523) and **SAMN28055956** (*R. subnigricans*, D338).

## Figures and Tables

**Table 1:** Genome features of six poisonous mushrooms under study.

Parameter	D346	D287	D392	D322	D523	D338
Genome coverage (x)	104	109	115	111	106	97
Number of contigs >1000 bp	13,343	4,607	8,758	14,820	9,798	13,345
Number of scaffolds >1000 bp	12,482	4,450	8,073	13,983	8,970	12,650
N50 of contigs (bp)	9,588	32,238	9,063	4,695	9,933	7,564
N50 of scaffolds (bp)	10,450	34,423	10,221	5,274	11,467	8,248
Longest scaffold length (kb)	259.8	674.9	800.6	103.4	117.1	92.6
**Total scaffold length (Mb)**	77.1	52.0	49.0	51.8	55.7	64.2
GC content (%)	44.9	43.5	43.5	46.2	39.7	49.0
Length of gap sequences (kb, ratio)	87.4, 0.1%	14.2, 0%	32.8, 0.1%	83.2, 0.2%	56.7, 0.1%	52.9, 0.1%
Number of genes predicted	8,181	7,353	8,808	8,592	5,375	14,099
Single-copy orthologs (%)	92.6	95.7	92.5	85.9	89.4	84.0
**Number of biosynthetic genes encoding**						
Indole synthase			1			
Nonribosomal peptide synthetase (NRPS)	2	2	2		1	2
Siderophore			2			1
Terpene synthase	7	8	16	5	6	8
Type I polyketide synthase (PKS)		1		1	1	
PKS-NRPS hybrid		2				
**Mitochondrial genome (bp)**	62,094	41,555	78,907	47,464	66,949	66,439
GC content (%)	23.4	21.5	31.9	25	27.3	21.6
